# Correction: Tissue-derived exosome proteomics identifies promising diagnostic biomarkers for esophageal cancer

**DOI:** 10.7554/eLife.95305

**Published:** 2023-12-20

**Authors:** Dingyu Rao, Hua Lu, Xiongwei Wang, Zhonghong Lai, Jiali Zhang, Zhixian Tang

**Keywords:** Human

Rao D, Lu H, Wang X, Lai Z, Zhang J, Tang Z. 2023. Tissue-derived exosome proteomics identifies promising diagnostic biomarkers for esophageal cancer. *eLife* **12**:e86209. doi: 10.7554/eLife.86209.

Published 15 November 2023

Prof. Zhixian Tang was originally mistakenly omitted from the author list, due to the authors not aware of the importance of Prof. Tang’s work and the role and significance of the corresponding author during the submission process. Zhixian Tang however he played a key role in this study including: the design of the experimental protocol; experimental implementation; the acquisition of esophageal tissue specimens; and the sequencing of the body mass profile of the derived esophageal tissue exosome from which the study target protein was derived. He also provided guidance with respect to the use of experimental technology and contributed to the financial support for this study. We are therefore formally correcting the eLife paper to include Zhixian Tang as the corresponding author on this paper.

After discussion with all authors of the article, including the new corresponding author (Zhixian Tang) and original co-corresponding authors Haibin Shen and Defa Huang, we have agreed to correct the eLife paper to remove both Haibin Shen and Defa Huang as authors since they no longer warrant authorship for the reasons that follow.

This clinical study mainly focused on the differential expression of exosomes in tissues and blood samples of patients with esophageal cancer and most of the work, such as the conception, experimental design, experimental implementation and article writing, were completed by the research group of Zhixian Tang. Although Haibin Shen and Defa Huang provided useful guidance on the extraction and identification of exosomes their contributions to the study do not warrant authorship and so their contributions have been added to the Acknowledgement section. All authors have agreed to the new author list, author contributions, have reviewed the manuscript and support the conclusions, and agree to be responsible for all parts of the paper in its published form.

**New authors list:**

Dingyu Rao, Hua Lu, Xiongwei Wang, Zhonghong Lai, Jiali Zhang, Zhixian Tang

**Original authors list:**

Dingyu Rao, Hua Lu, Xiongwei Wang, Zhonghong Lai, Jiali Zhang, Haibin Shen, Defa Huang

**Details for the omitted author:**

Zhixian Tang

Department of Cardiothoracic Surgery, First Affiliated Hospital of Gannan Medical University, Ganzhou, China;

**For correspondence:** tangzhixian@gmu.cn

**Contribution:** Data curation, Formal analysis, Investigation, Conceptualization, Validation

**Competing Interests:**

The authors declare no conflicts of interest.

**Details of the two removed (previously co-corresponding) authors:**

Haibin Shen

Laboratory Medicine, First Affiliated Hospital of Gannan Medical University, Ganzhou, China

Defa Huang

Laboratory Medicine, First Affiliated Hospital of Gannan Medical University, Ganzhou, China

**Acknowledgements:**

We thank Dr. Huang and Dr. Shen of Laboratory Medicine, the First Affiliated Hospital of Gannan Medical University for providing technical guidance and support.

The article has been corrected accordingly.

