# Supplementary figures and images for: Tissue-derived exosome proteomics identifies promising diagnostic biomarkers for esophageal cancer
Source: eLife. 2023 Nov 15;12:e86209. doi: 10.7554/eLife.86209 (PMC10651172; doi:10.7554/eLife.86209)

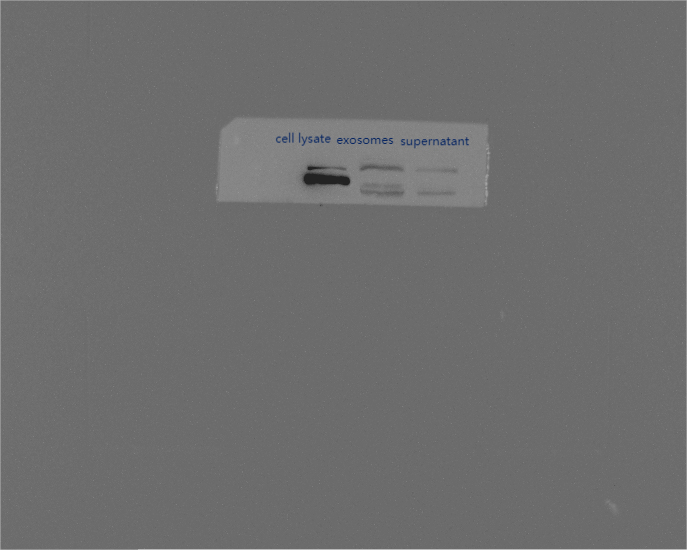

Supplement: Figure 1—source data 1. [file elife-86209-fig1-data1.zip › Figure 1D┬í┬¬source data/TSG101.jpg]

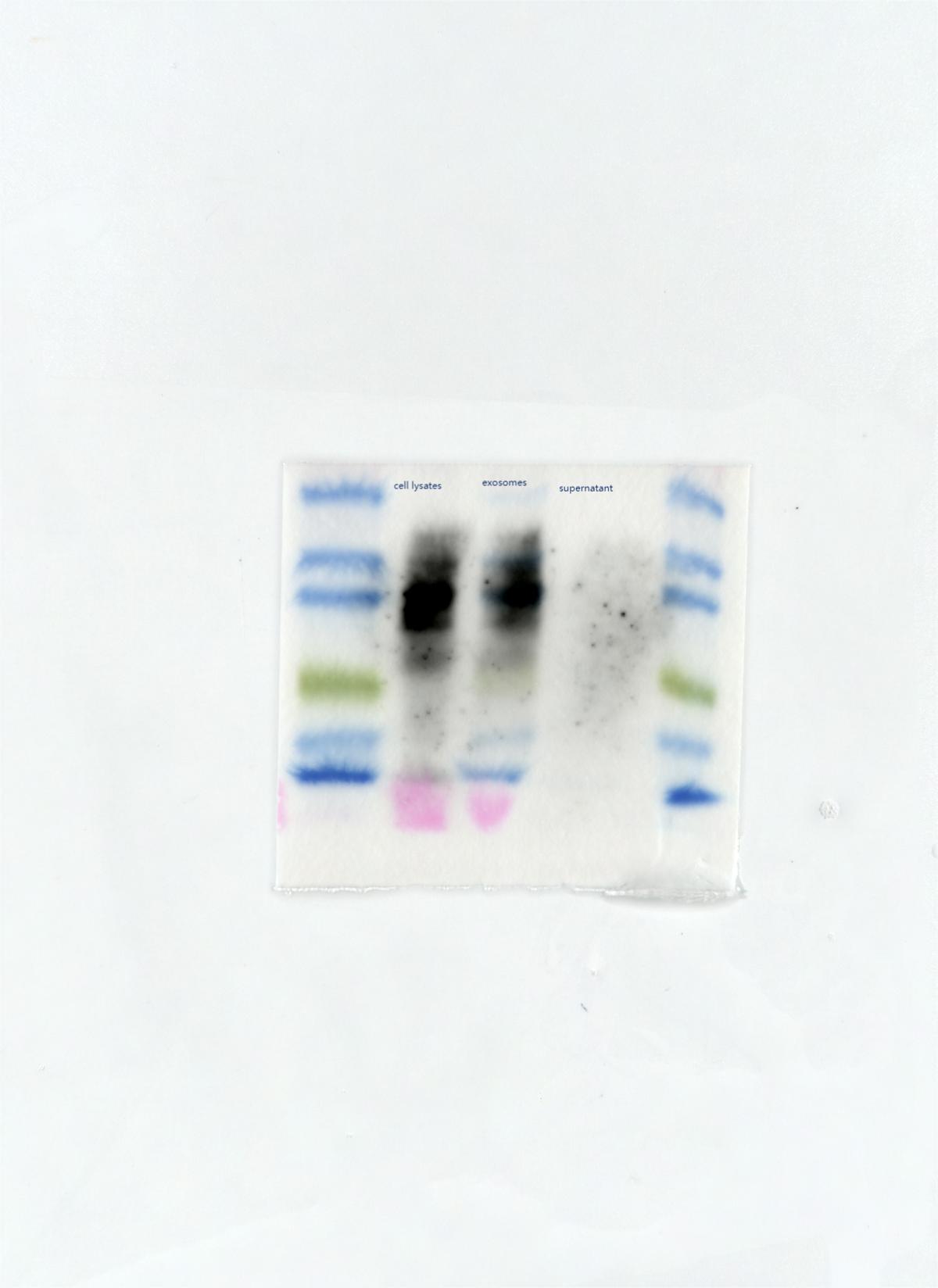

Supplement: Figure 1—source data 1. [file elife-86209-fig1-data1.zip › Figure 1D┬í┬¬source data/CD63.jpg]

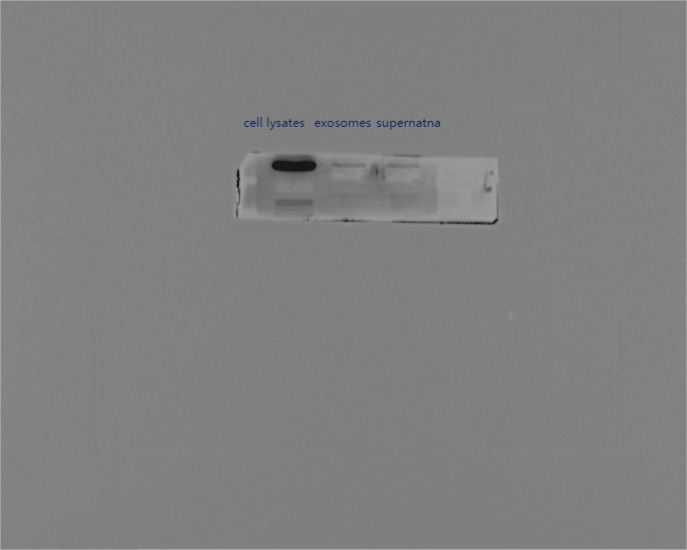

Supplement: Figure 1—source data 1. [file elife-86209-fig1-data1.zip › Figure 1D┬í┬¬source data/GM130.jpg]

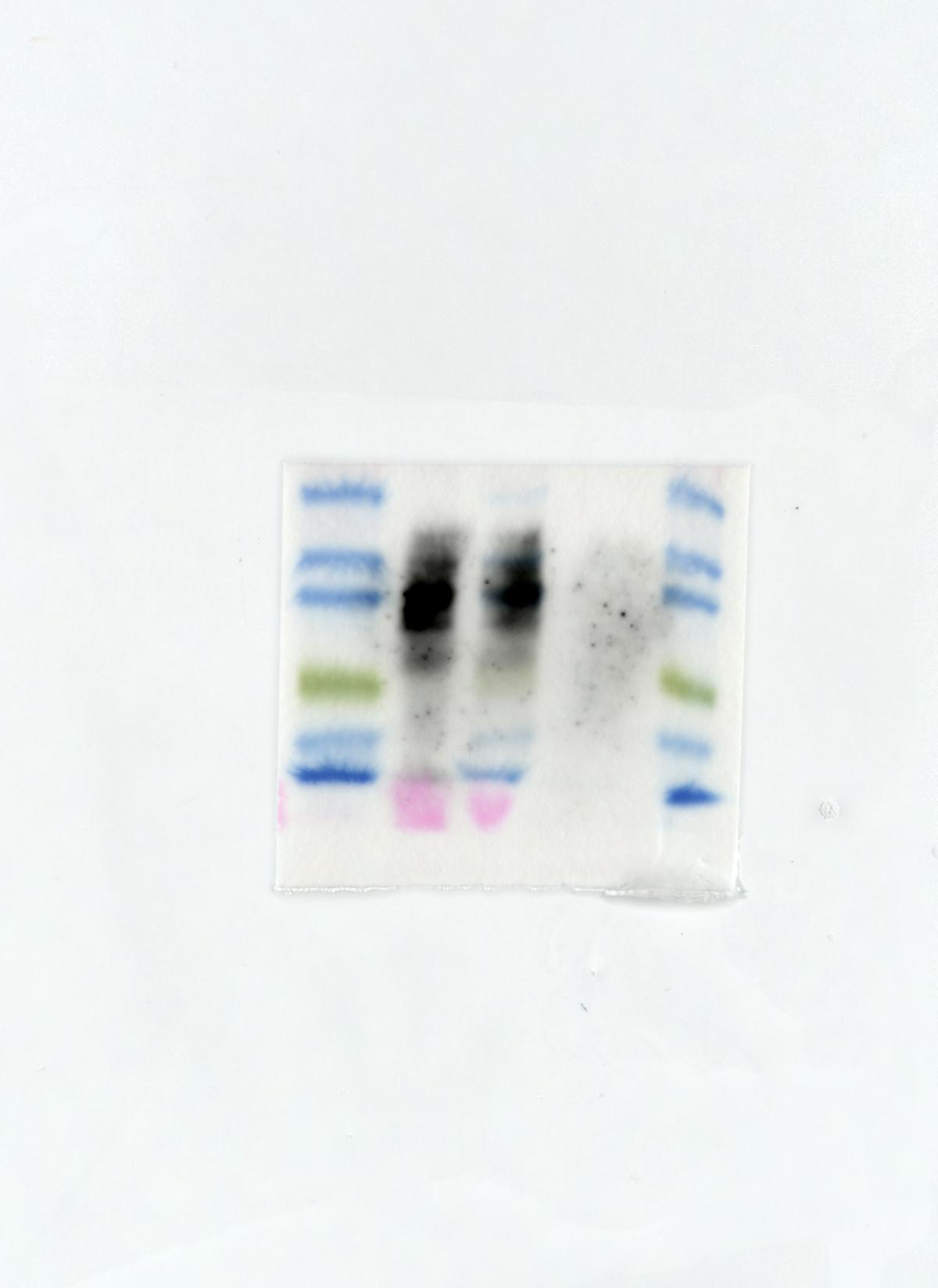

Supplement: Figure 1—source data 1. [file elife-86209-fig1-data1.zip › Figure 1D┬í┬¬source data/CD63.tif]

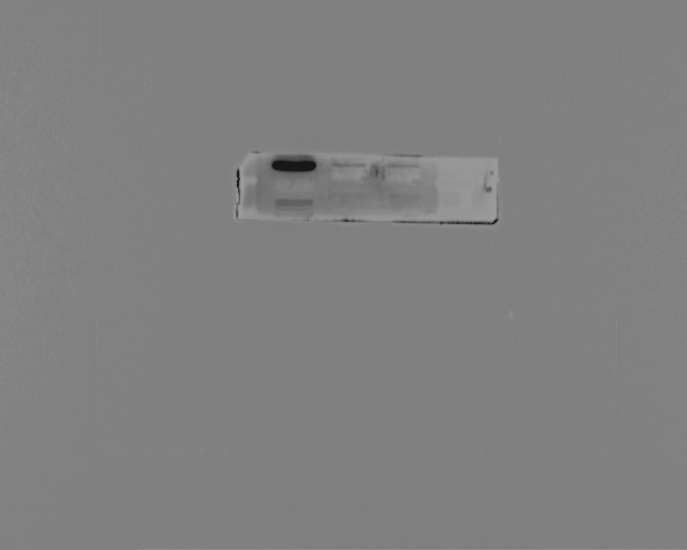

Supplement: Figure 1—source data 1. [file elife-86209-fig1-data1.zip › Figure 1D┬í┬¬source data/GM130.tif]

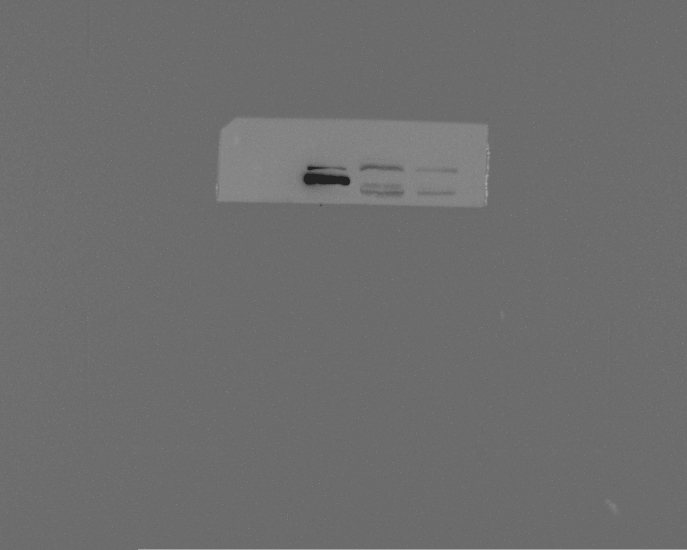

Supplement: Figure 1—source data 1. [file elife-86209-fig1-data1.zip › Figure 1D┬í┬¬source data/TSG101.tif]

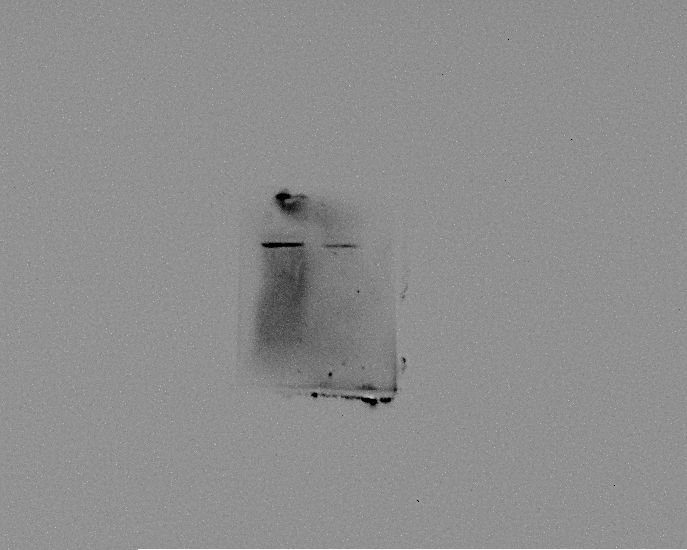

Supplement: Figure 1—source data 1. [file elife-86209-fig1-data1.zip › Figure 1D┬í┬¬source data/CD9.tif]

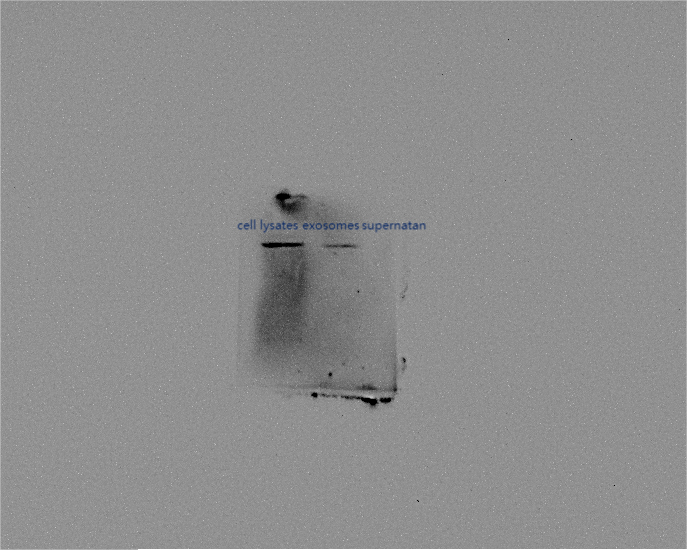

Supplement: Figure 1—source data 1. [file elife-86209-fig1-data1.zip › Figure 1D┬í┬¬source data/CD9.jpg]

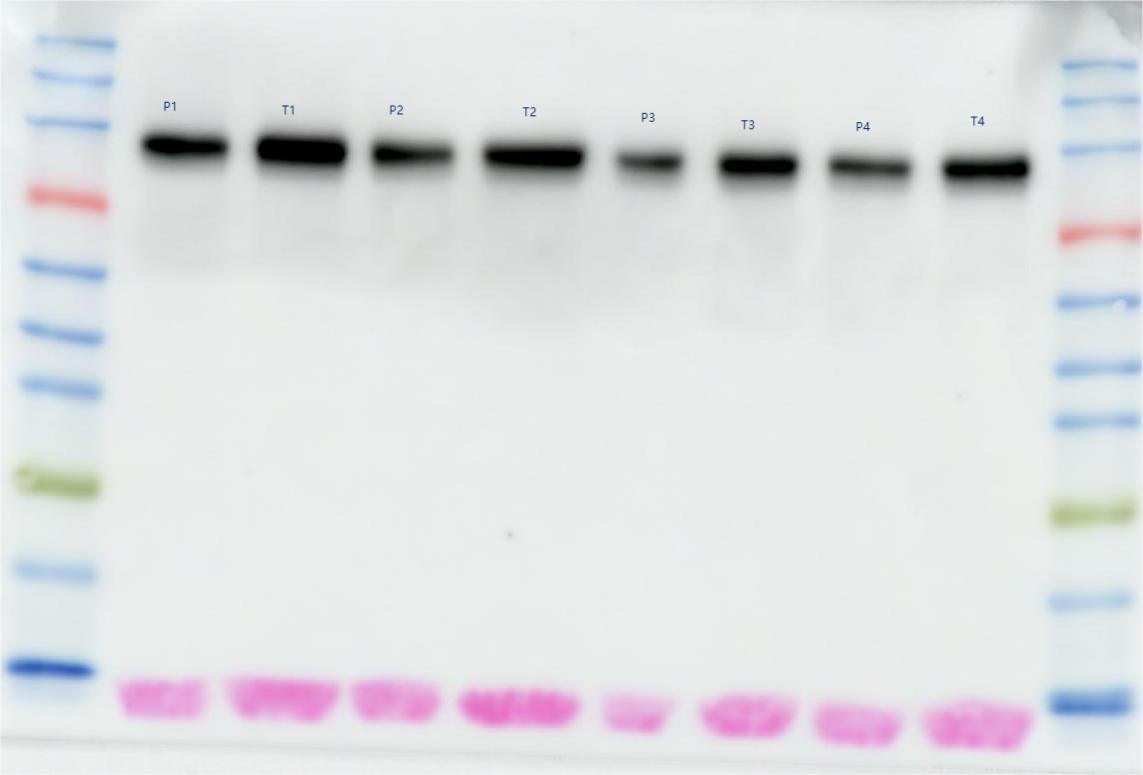

Supplement: Figure 4—source data 1. [file elife-86209-fig4-data1.zip › Figure 4C┬í┬¬source data/CD54-1.jpg]

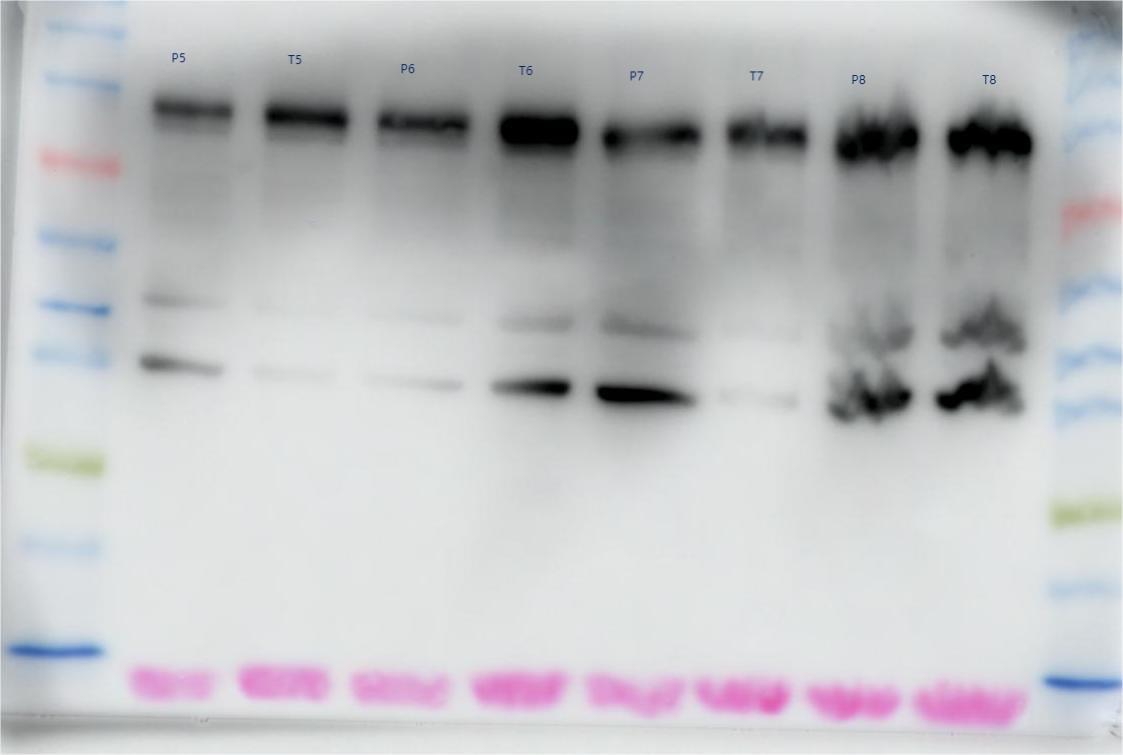

Supplement: Figure 4—source data 1. [file elife-86209-fig4-data1.zip › Figure 4C┬í┬¬source data/CD54-2.jpg]

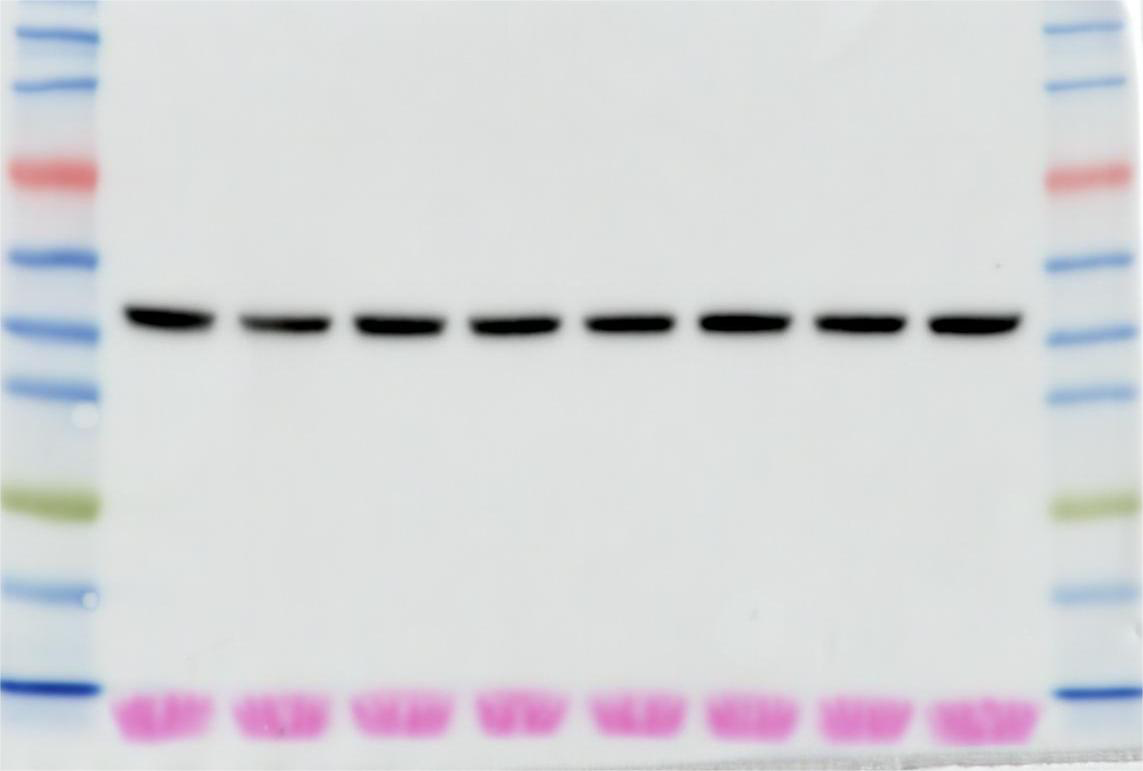

Supplement: Figure 4—source data 1. [file elife-86209-fig4-data1.zip › Figure 4C┬í┬¬source data/Actin-1.tif]

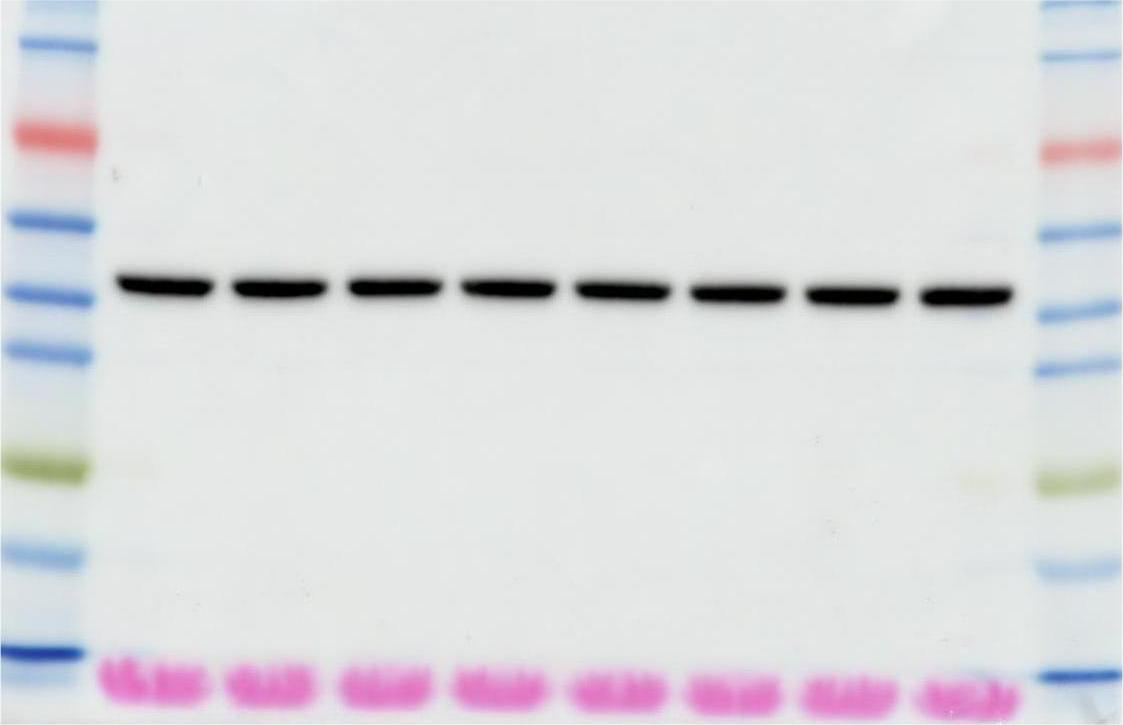

Supplement: Figure 4—source data 1. [file elife-86209-fig4-data1.zip › Figure 4C┬í┬¬source data/Actin-2.tif]

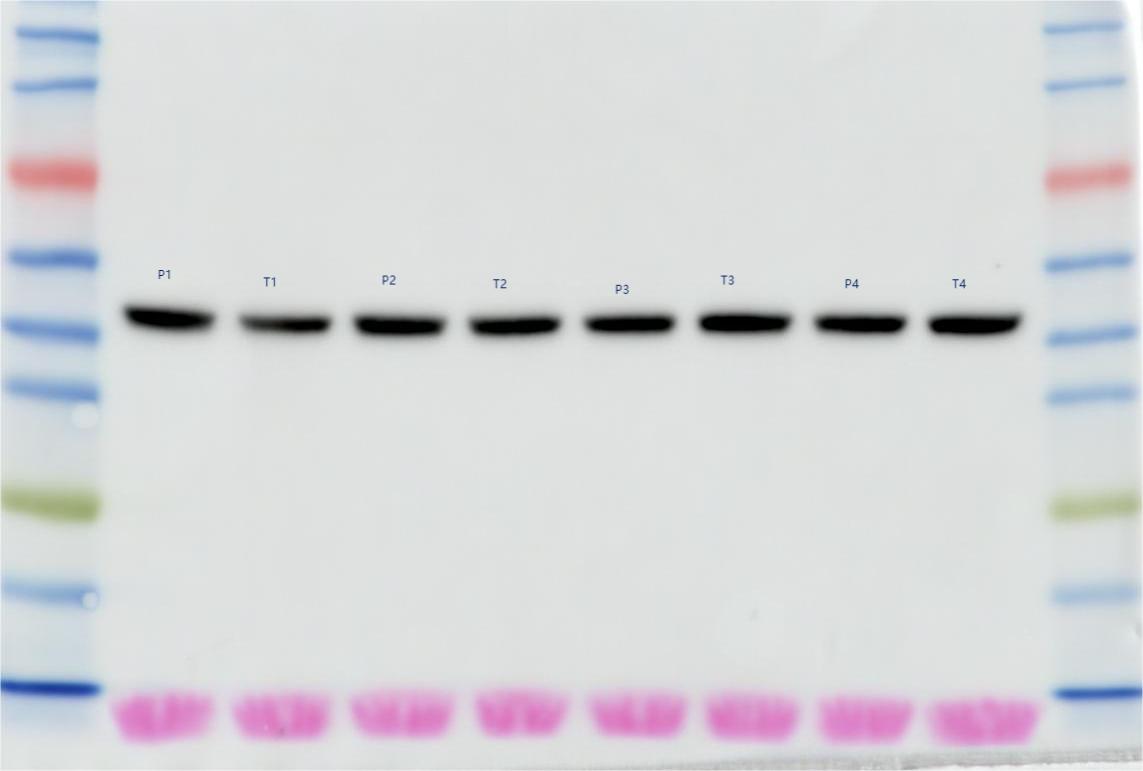

Supplement: Figure 4—source data 1. [file elife-86209-fig4-data1.zip › Figure 4C┬í┬¬source data/Actin-1.jpg]

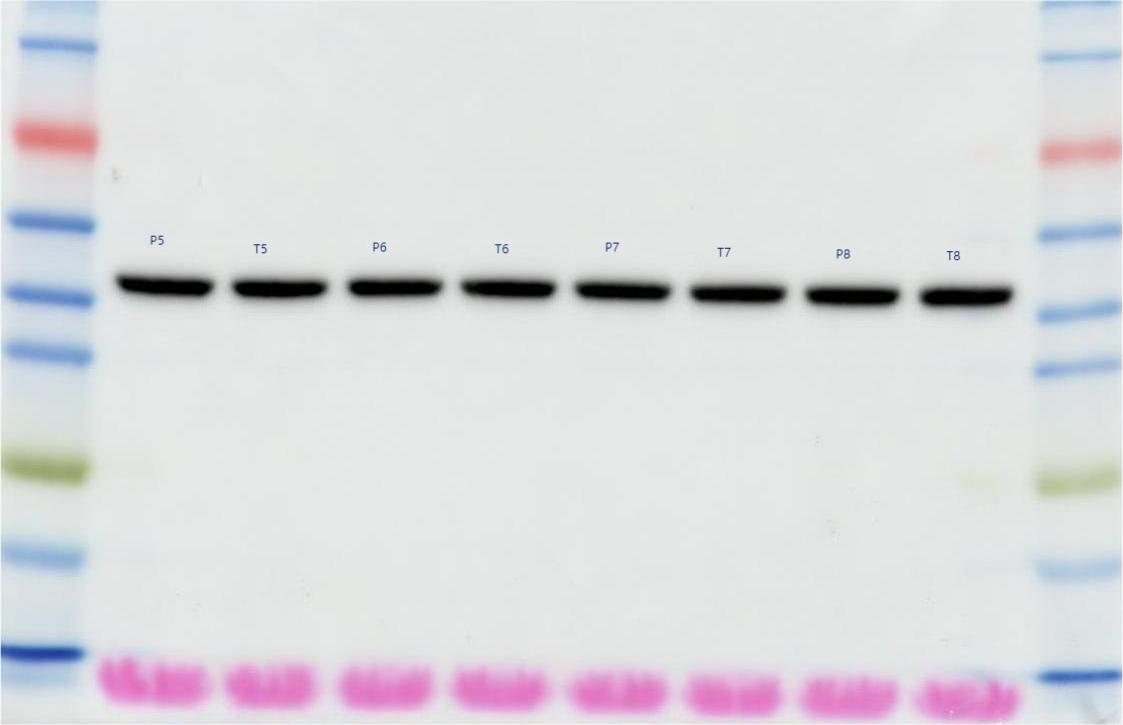

Supplement: Figure 4—source data 1. [file elife-86209-fig4-data1.zip › Figure 4C┬í┬¬source data/Actin-2.jpg]

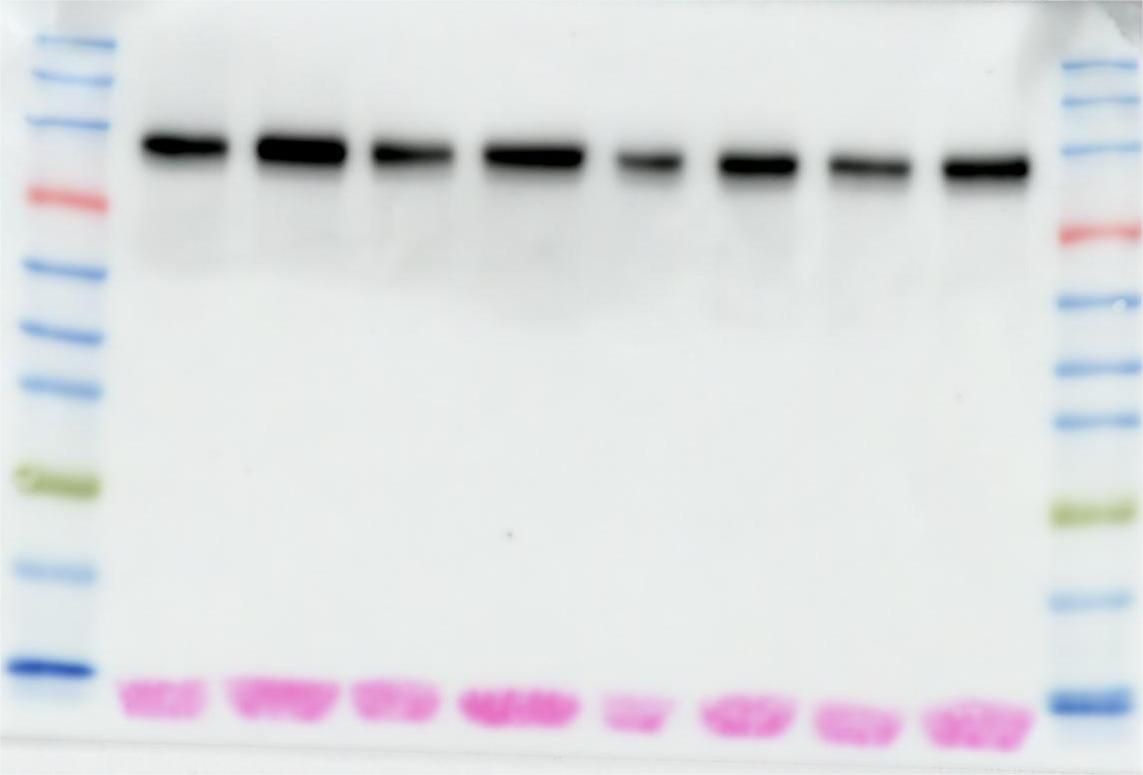

Supplement: Figure 4—source data 1. [file elife-86209-fig4-data1.zip › Figure 4C┬í┬¬source data/CD54-1.tif]

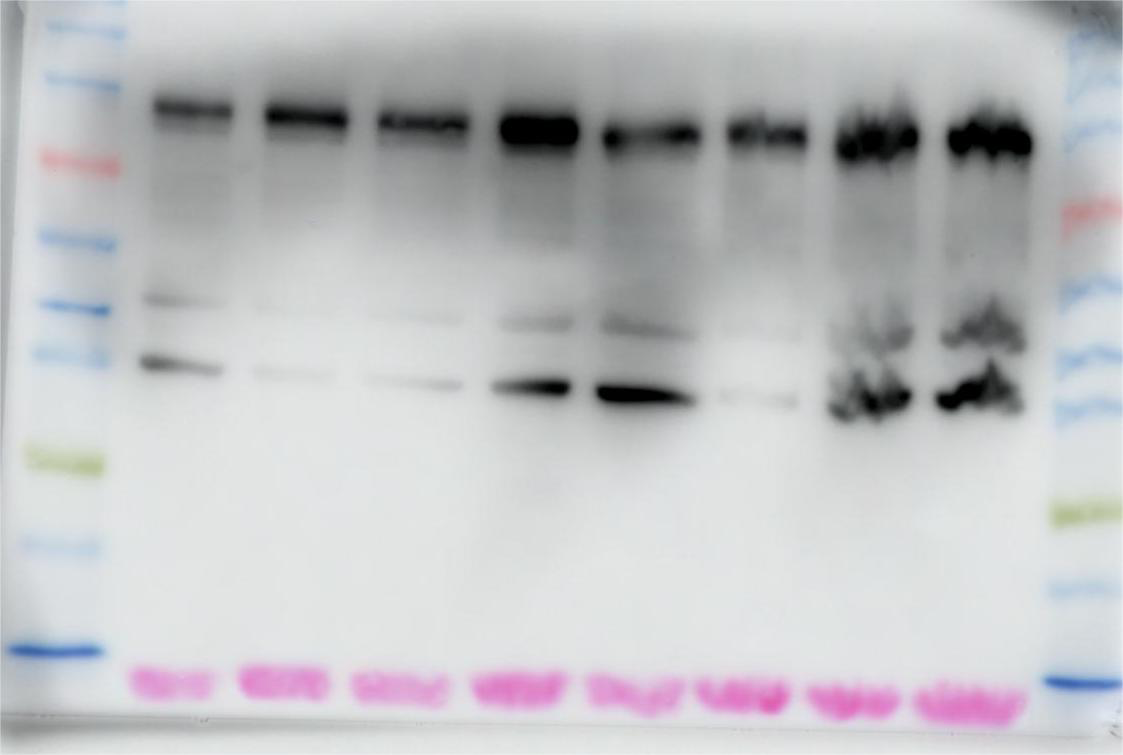

Supplement: Figure 4—source data 1. [file elife-86209-fig4-data1.zip › Figure 4C┬í┬¬source data/CD54-2.tif]

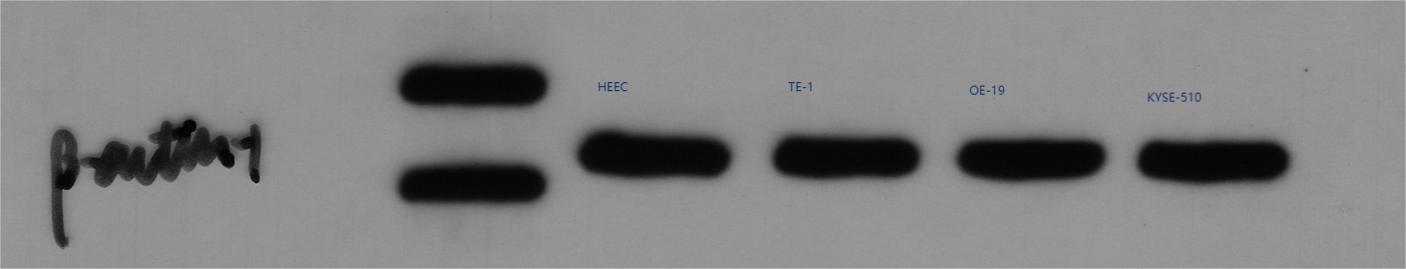

Supplement: Figure 8—source data 1. [file elife-86209-fig8-data1.zip › Figure 8B┬í┬¬source data/actin.jpg]

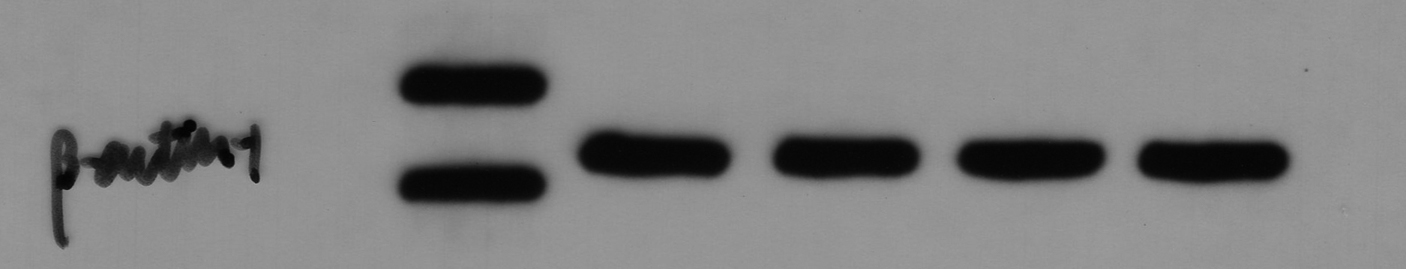

Supplement: Figure 8—source data 1. [file elife-86209-fig8-data1.zip › Figure 8B┬í┬¬source data/actin.tif]

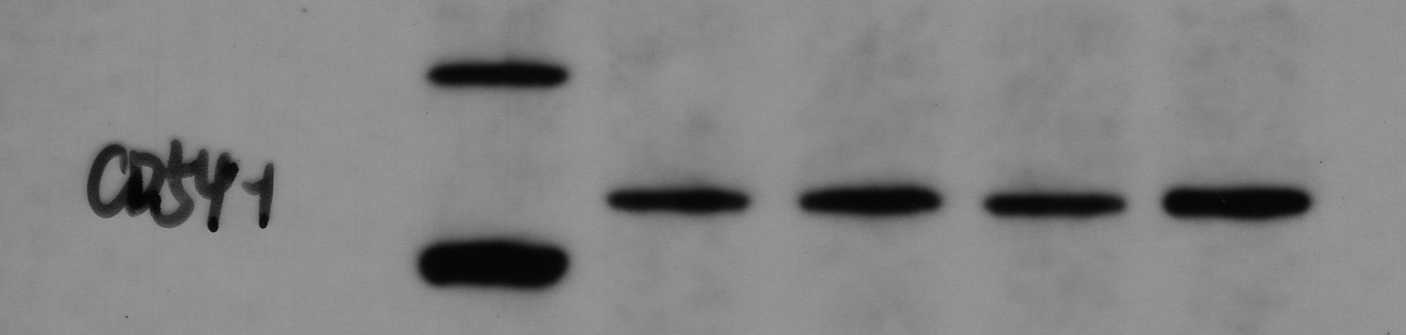

Supplement: Figure 8—source data 1. [file elife-86209-fig8-data1.zip › Figure 8B┬í┬¬source data/CD54.tif]

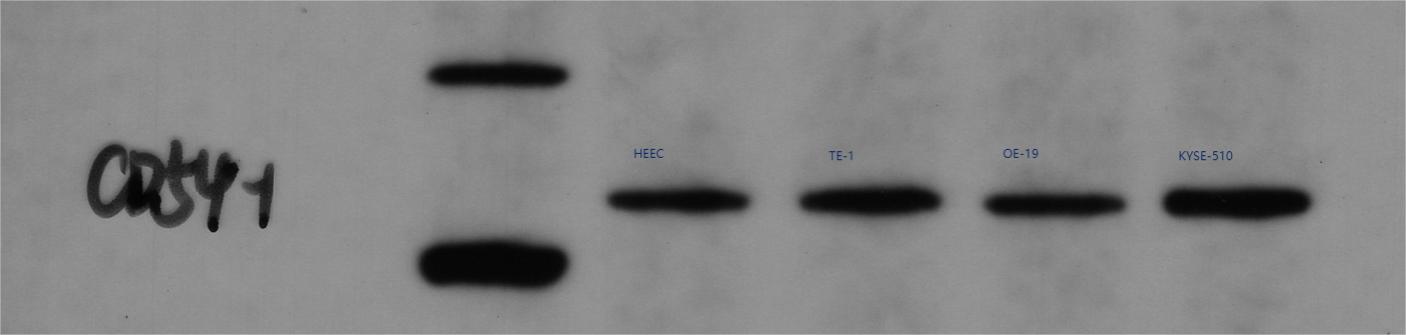

Supplement: Figure 8—source data 1. [file elife-86209-fig8-data1.zip › Figure 8B┬í┬¬source data/CD54.jpg]

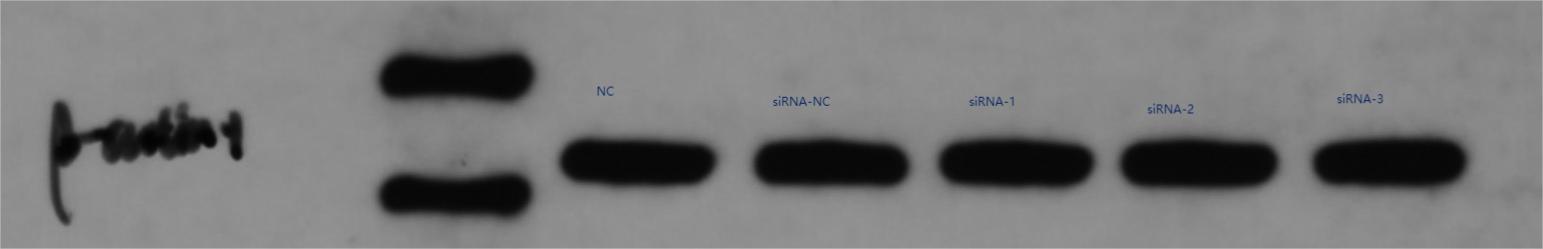

Supplement: Figure 8—source data 2. [file elife-86209-fig8-data2.zip › Figure 8E┬í┬¬source data/actin.jpg]

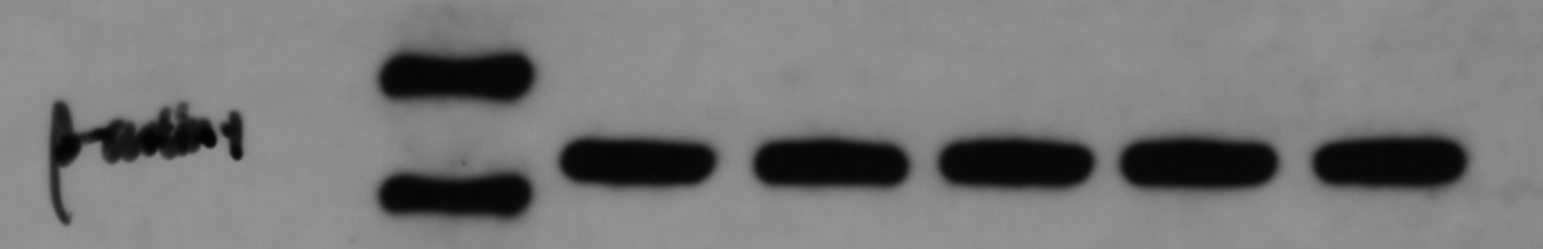

Supplement: Figure 8—source data 2. [file elife-86209-fig8-data2.zip › Figure 8E┬í┬¬source data/actin.tif]

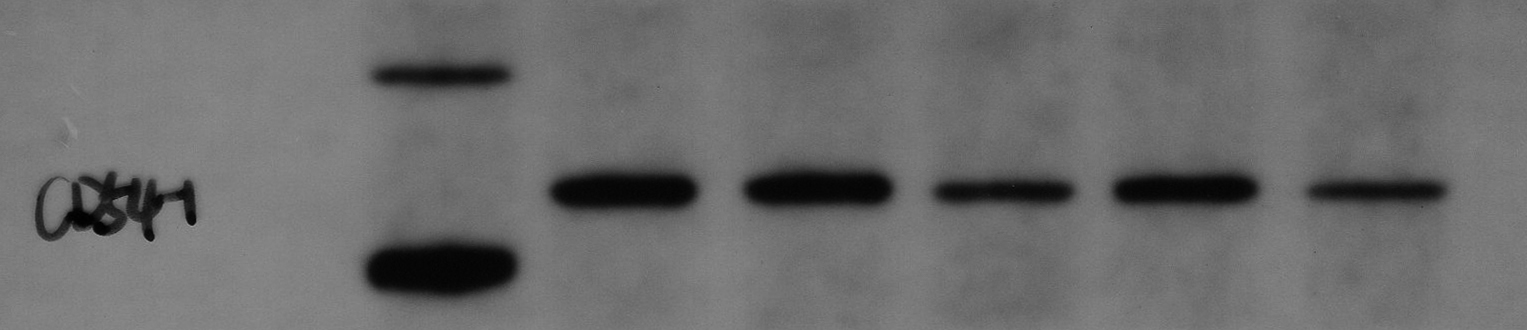

Supplement: Figure 8—source data 2. [file elife-86209-fig8-data2.zip › Figure 8E┬í┬¬source data/CD54.tif]

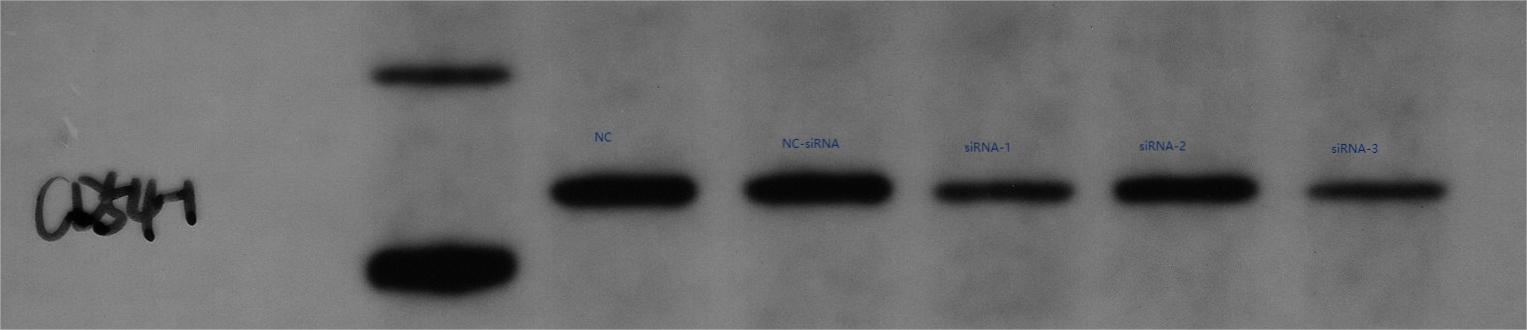

Supplement: Figure 8—source data 2. [file elife-86209-fig8-data2.zip › Figure 8E┬í┬¬source data/CD54.jpg]
